# Effects of Physical Activity Level on Microsaccade Dynamics During Optic Flow Stimulation in Adults with Type 2 Diabetes

**DOI:** 10.3390/biomedicines14010231

**Published:** 2026-01-21

**Authors:** Milena Raffi, Alessandra Laffi, Andrea Meoni, Michela Persiani, Lucia Brodosi, Alba Nicastri, Maria Letizia Petroni, Alessandro Piras

**Affiliations:** 1 Department of Biomedical and Neuromotor Sciences DIBINEM, Bologna University, 40126 Bologna, Italy; alessandra.laffi3@unibo.it (A.L.); andrea.meoni@unibo.it (A.M.); michela.persiani3@unibo.it (M.P.); 2 Clinical Nutrition and Metabolism Unit, Policlinico S. Orsola, Azienda Ospedaliero-Universitaria di Bologna, 40138 Bologna, Italy; lucia.brodosi2@unibo.it; 3 Department of Medical and Surgical Sciences, Bologna University, 40138 Bologna, Italy; alba.nicastri@studio.unibo.it (A.N.); marialetizia.petroni@unibo.it (M.L.P.); 4 Department of Quality of Life Studies QUVI, Bologna University, 47921 Rimini, Italy; alessandro.piras3@unibo.it

**Keywords:** microsaccades, eye movements, diabetes, physical exercise, physical activity

## Abstract

**Background**: Microsaccades are small fixational eye movements tightly linked to attention and oculomotor control. Although diabetes mellitus is associated with retinal and neural alterations that may impair visuomotor function, the influence of physical activity on microsaccade behaviour in individuals with type 2 diabetes mellitus (T2DM) remains unknown. This study investigated whether habitual physical activity modulates microsaccade characteristics during fixation under different optic flow stimuli. Given that optic flow engages motion processing and gaze stabilisation pathways that may be affected by diabetes-related microvascular/neural changes, it can reveal subtle visuomotor alterations during fixation. **Methods**: Twenty-eight adults with T2DM and no diagnosed retinopathy performed a fixation task while viewing optic flow stimuli made of moving dots. Eye movements were recorded using an EyeLink system. Physical activity behaviour was assessed at baseline and at a 6-month follow-up after a low-threshold aerobic circuit training programme. Classification as physically active (≥600 MET-min/week) or inactive (<600 MET-min/week) was based on the 6-month assessment. Microsaccade characteristics were analysed by repeated-measures ANOVA. **Results**: Microsaccade rate was modulated by optic flow (*p* = 0.044, η^2^p = 0.106) and showed a significant stimulus × group × sex interaction (*p* = 0.005, η^2^p = 0.163), indicating sex-dependent differences in how optic flow modulated microsaccade rate across physically active and inactive participants. A time × stimulus interaction effect was found in peak velocity (*p* = 0.03, η^2^p = 0.114) and amplitude (*p* = 0.02, η^2^p = 0.127), consistent with modest context-dependent changes over time. **Conclusions**: These findings suggest that physical activity modulates microsaccade generation and supports the potential of microsaccade metrics as sensitive indicators of oculomotor function in diabetes.

## 1. Introduction

Even during attempted steady fixation on a visual target, the eyes are never still; they continually execute small involuntary movements, including slow drifts, tremors, and microsaccades, which are rapid and small-amplitude eye movements occurring during fixation. Microsaccade characteristics include a low frequency, typically ~1–3 per second, a low amplitude, at less than 1°, and a peak velocity of up to 100°/s [1,2]. Microsaccades serve several key visual functions, refreshing the retinal image to prevent perceptual fading and optimising the sampling of fine spatial detail by repositioning the fovea [3]. Beyond low-level stabilisation functions, several studies show that microsaccades are closely linked to attention and oculomotor control, given that their rate, amplitude, directional bias, and temporal modulation reflect those of attention deployment, cognitive state, and readiness for action [4,5,6]. Microsaccades are also sensitive indicators of attentional dynamics [7], being biassed toward the locus of covert attention [8]. This close coupling between microsaccades and attentional processes highlights their potential as noninvasive markers of visual and cognitive control mechanisms relevant to oculomotor and visuomotor function.

Diabetes mellitus is a chronic metabolic disease often characterised by complications, including microvascular damage, neuropathy, and retinal disease such as diabetic macular edoema and diabetic retinopathy. Visual and oculomotor systems are vulnerable in diabetes; fixation stability is poorer in macular disease, and fixational saccade amplitude and area of fixation are larger in diabetic maculopathy [9]. Visuomotor deficits are a common complication of diabetes, particularly in the presence of retinal or neural alterations. Such deficits are expected to manifest at the level of microsaccade metrics, for example, through changes in microsaccade rate, amplitude, or temporal modulation during fixation. On this basis, microsaccades provide a mechanistically grounded window into fine alterations of visuomotor control in individuals with diabetes.

Physical activity and structured exercise are widely recognised to influence systemic health, such as metabolic control, vascular function, and neural plasticity, as well as cognitive–motor control like attention, executive function, oculomotor readiness. In sports and vision sciences, individuals with higher training and fitness status show differences in attention metrics and oculomotor behaviour compared with less active individuals [10,11,12]. Tsai et al. [13] demonstrated that a 30 min bout of moderate or high-intensity aerobic exercise significantly modulated post-exercise antisaccade control and altered saccadic peak velocity in middle-aged and older adults. Earlier work found that a 10 min bout of moderate–vigorous aerobic exercise improved executive-related oculomotor control (e.g., antisaccade performance), likely via increased attention, arousal, and engagement of frontoparietal networks that also underpin gaze control [14]. Although the direct literature on microsaccades and exercise is still modest, the mechanistic links are strong: increased arousal and attention, improved sensorimotor coordination, and enhanced oculomotor control via training all could modulate microsaccade behaviour.

Given that microsaccades are sensitive to both attentional and oculomotor states, it is possible to hypothesise that individuals with higher physical activity level might exhibit a different microsaccade behaviour due to enhanced attentional-oculomotor integration. Despite these converging aspects, such as microsaccades as biomarkers of attention and/or oculomotor control, diabetes as a condition with documented oculomotor alterations, and physical activity as a modulator of attention and oculomotor systems, there is a compelling gap in the literature. To our knowledge, the relationship between physical activity or exercise interventions and microsaccade generation during fixation in people with diabetes has not yet been systematically investigated. Filling this gap would provide both theoretical and practical promise. Theoretically, it would link systemic behavioural modulation (exercise) to oculomotor micro-control (microsaccades) in a clinical population. Practically, it could offer a novel sensitive outcome metric for interventions and help illuminate mechanisms of visual/oculomotor dysfunction in diabetes.

The perception of optic flow, the coherent motion pattern generated during self-motion, engages complex neural mechanisms that integrate sensory input, body position in space, and oculomotor feedback. The optic flow fields are crucial for navigation in the environment, as they provide continuous visual information about self-motion and spatial orientation [15,16,17]. The analysis of optic flow contributes to the coordination of postural stability, heading perception, and motor planning, enabling precise adjustments of motor activity and eye movements [18,19,20]. Oculomotor activity, including fixational and compensatory eye movements, plays a central role in maintaining visual stability and extracting relevant motion cues from optic flow fields. Alterations in these mechanisms can therefore impair balance and spatial orientation, particularly in people with diabetes, where retinal and neural changes may affect the processing of optic flow and the control of gaze [21,22]. Investigating eye movement behaviour during optic flow stimulation may therefore indicate deficits in visuomotor integration associated with diabetes, characterising functional changes that are not evident through standard clinical measures.

Despite extensive evidence linking microsaccades to attentional and oculomotor control, and well-documented visuomotor alterations in diabetes, no study, to our knowledge, has directly examined whether habitual physical activity modulates microsaccade dynamics in individuals with type 2 diabetes mellitus (T2DM), particularly under optic flow stimulation. Moreover, optic flow represents a demanding visuomotor context that may reveal tiny functional alterations not detectable during simple fixation.

The aim of the present study is to examine the influence of physical activity level on microsaccade generation during the viewing of optic flow stimuli in individuals with T2DM. We assessed the variability of microsaccade rate, amplitude, peak velocity, and duration during a controlled fixation paradigm and related these metrics to the physical activity status of the participants. We hypothesised that physically active and inactive individuals would show differences in microsaccade dynamics, and that these differences would be modulated by optic flow conditions, reflecting activity-related variations in visuomotor and attentional control.

## 2. Materials and Methods

For the realisation of this study, we enrolled 28 individuals with T2DM with no diagnosis of diabetic retinopathy or diabetic macular edoema. Inclusion criteria required normal or corrected-to-normal visual acuity sufficient to perform the fixation task, absence of diagnosed neurological or ophthalmological disorders other than diabetes, and the ability to maintain stable fixation during eye-tracking calibration. Participants with a history of major neurological disease, vestibular disorders, or ocular conditions known to affect fixation stability were excluded. All participants were under stable medical treatment for diabetes at the time of testing; no changes in medication were reported during the study period. Before the beginning of the experiment, each participant signed a written informed consent form. The study protocol was approved by the Institutional Bioethic Committee of the University of Bologna (Prot. n. 0283851, approval date 4 November 2021). The experiments were performed in accordance with the ethical standards laid down in the 1964 Declaration of Helsinki.

At baseline, all participants reported no engagement in structured physical activity. According to the World Health Organization, the minimum recommended level of aerobic physical activity for adults corresponds to at least 150–300 min per week of moderate-intensity activity, or 75–150 min per week of vigorous-intensity activity, or an equivalent combination of the two [23]. Accordingly, physical inactivity was operationally defined as failure to meet these minimum recommended thresholds. To address behavioural and physiological barriers to regular exercise commonly observed in individuals with T2DM, the intervention was implemented as a 6-month standardised, low-threshold aerobic circuit training programme. Aerobic exercise, including circuit-based modalities, is consistently recommended in clinical practice for patients with type 2 diabetes due to its established benefits on cardiorespiratory fitness, insulin sensitivity, and glycaemic control [24]. Training sessions were delivered either as supervised land-based aerobic circuit training in a gym or as structured aquatic cardio-circuit sessions in a swimming pool, an option chosen to enhance feasibility for participants with overweight or obesity, joint discomfort, or reduced mobility. No resistance or load-based training modalities were included, and a single, homogeneous exercise modality was maintained throughout the intervention to ensure methodological consistency. Both land-based and aquatic training were designed to provide a comparable aerobic stimulus in terms of exercise intensity and duration, in line with clinical exercise recommendations for individuals with T2DM. The use of two training environments was motivated by feasibility considerations, rather than by an expectation of differential physiological or oculomotor effects. Accordingly, no mode-specific effects were hypothesised or analysed in the present study.

Physical activity behaviour was assessed at a single 6-month follow-up through a structured face-to-face interview conducted by trained researchers. Physical activity was assessed using the International Physical Activity Questionnaire (IPAQ) [25], a widely used and validated instrument in clinical and metabolic populations, including individuals with type 2 diabetes. Although self-reported measures may be subject to recall bias, IPAQ allows standardised classification of physical activity levels in applied and clinical settings and was selected for its feasibility within the present intervention context.

Each reported activity was assigned a metabolic equivalent of task (MET) value based on the Adult Compendium of Physical Activities [26]. Weekly energy expenditure was calculated following the standard procedure indicated in the IPAQ guidelines:

MET-min/week = MET value × minutes of activity × days per week

**Figure 1:**
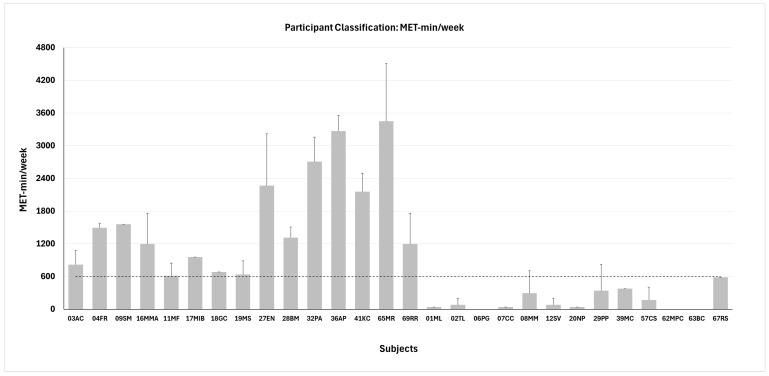
Participant classification in active (*n* = 15, left side) and inactive (*n* = 13, right side) groups based on the metabolic equivalent (MET) of task. Dashed line represents the threshold for classification. Data are shown as mean values ± SD for each participant.

The reference threshold for classification was established according to the IPAQ Research Committee (2005) [25], which defines the cut-off for distinguishing between physically active and physically inactive individuals at 600 MET-min/week. Accordingly, participants accumulating ≥600 MET-min/week at the 6-month follow-up were classified as physically active (*n* = 15; age 57.3 ± 10.0 years; BMI 29.0 ± 5.7 kg/m^2^; HbA1c 55.1 ± 19.2 mmol/mol), whereas those accumulating <600 MET-min/week were classified as physically inactive (*n* = 13; age 60.2 ± 11.7 years; BMI 32.8 ± 5.0 kg/m^2^; HbA1c 52.5 ± 10.4 mmol/mol). The mean value ± SD for each participant is shown in Figure 1.

### 2.1. Optic Flow Stimuli

**Figure 2:**
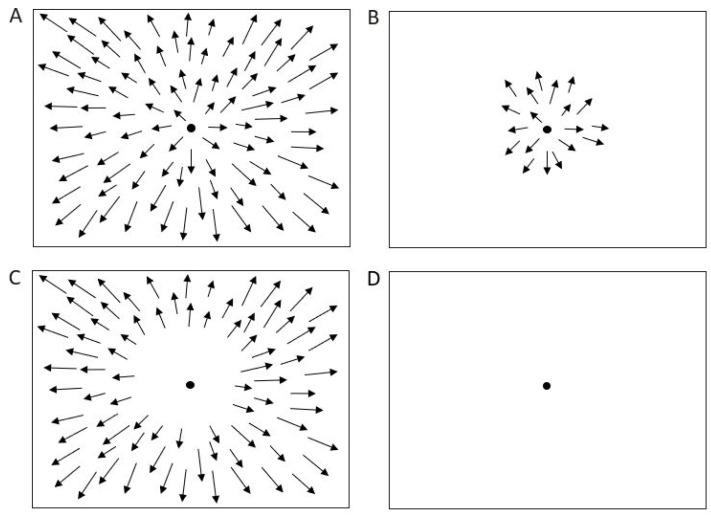
Optic flow and control stimuli. (**A**) Full field stimulation. (**B**) Foveal stimulation. (**C**) Peripheral stimulation. (**D**) Baseline condition (control). Arrows represent the velocity vectors of moving dots.

In this experiment, we presented three types of optic flow stimuli designed to activate different regions of the visual field: full-field, foveal, and peripheral (Figure 2A–C). Foveal stimuli originated from the focus of expansion, being shown only in the central 7°, while the entire peripheral area was occluded. Peripheral stimuli covered the entire screen, except a central occlusion circle of 20° in radius [19].

Each stimulus consisted of white dots (luminance = 1.3 cd/m^2^; size = 0.4°), moving at a speed of 5°/s, projected onto a translucent screen subtending at a visual angle of 135 × 107°. The stimuli were back-projected in a dark room with non-reflective walls. Participants were instructed to maintain fixation on a 0.6° fixation point (FP) without head movements; the screen height was individually adjusted so that the FP aligned with the participant’s primary gaze position. Viewing distance was fixed at 115 cm for all subjects.

A control condition consisted of simple fixation on a dark screen (Figure 2D). Optic flow stimuli were generated using the Psychophysics Toolbox for MATLAB (The MathWorks Inc., Natick, MA, USA).

Each participant completed two repetitions of both the baseline and the optic flow conditions, yielding a total of 8 trials per subject. Each trial lasted 30 s.

### 2.2. Eye Movement and Eye Position Recordings

The horizontal and vertical eye movements were recorded using the EyeLink eye tracking system (EyeLink^®^ II, SR Research Ltd., Mississauga, ON, Canada) consisting of two miniature cameras mounted on a leather-padded headband. Oculomotor recordings were performed before and after the 6 months of intervention. Pupil tracking was recorded at a sample frequency of 500 Hz, with high spatial resolution (<0.005°) and low noise (<0.01°). The calibration was performed at the beginning of each recording session; the participants were instructed to fixate a target presented in a random order in a nine-point 25 × 25° square grid. We repeated the calibration procedure until achieving a suitable score (average error < 0.5°, maximum error < 1.0°). After the camera was calibrated, the data were validated, and drift correction was performed by applying a corrective offset to the raw eye position.

Each participant completed eight trials of 30 s duration, yielding several hundred microsaccades per individual across conditions. In microsaccade research, statistical robustness is primarily driven by the number of detected events rather than by the number of trials per se, and trial numbers comparable to or lower than those used here have been shown to provide stable estimates of microsaccade metrics in fixation and optic flow paradigms. The present design also balanced data quantity with participant comfort and fixation quality, as previously adopted in similar microsaccade and optic flow studies [18,19,20].

### 2.3. Data Analysis

Microsaccades are small eye movements that occur during extended visual fixation with an amplitude of less than 1° [27]. To identify microsaccades, we developed an algorithm, already used in previous studies [20] based on that of Otero-Millan [28]. To minimise potential noise, we included only binocular microsaccades that were detected for at least three consecutive data samples (6 ms). Trials containing inaccurate fixations, eye blinks, or behavioural errors were excluded from analysis. Segments showing abrupt changes in pupil area exceeding 50 units per sample were also discarded, as such variations likely reflected partial (semi-)blinks during which the pupil was not fully visible. In addition, the 200 ms preceding and following each blink or semi-blink were removed to exclude periods when the pupil was still partially occluded [29].

For each subject, trial, and condition, we calculated the amplitude, duration, latency, rate, and peak velocity of the detected microsaccades. These parameters were then averaged across subjects for each condition and trial. Microsaccade rate was computed considering only the time spent in fixation: the total number of microsaccades in a given trial was divided by the total fixation time for that trial. Microsaccade latency was determined by averaging the onset latencies of all microsaccades relative to the beginning of each trial.

Statistical analysis was performed using a repeated-measures ANOVA with stimuli (foveal, full field, periphery) and time (baseline, 6 months) as within-subject factors, while group (active, inactive) and sex were between-subject factors (SPSS^®^ 22.0, Chicago, IL, USA). Partial eta squared (η^2^p) was used for effect size estimation, with small (~0.01), medium (~0.06), and large (≥0.14) effects [30]. Statistical significance was set at *p* < 0.05. Post hoc testing was corrected using the Bonferroni procedure.

## 3. Results

The data analysis showed significant effects in the microsaccade rate, amplitude, and peak velocity.

### 3.1. Main Sequence

**Figure 3:**
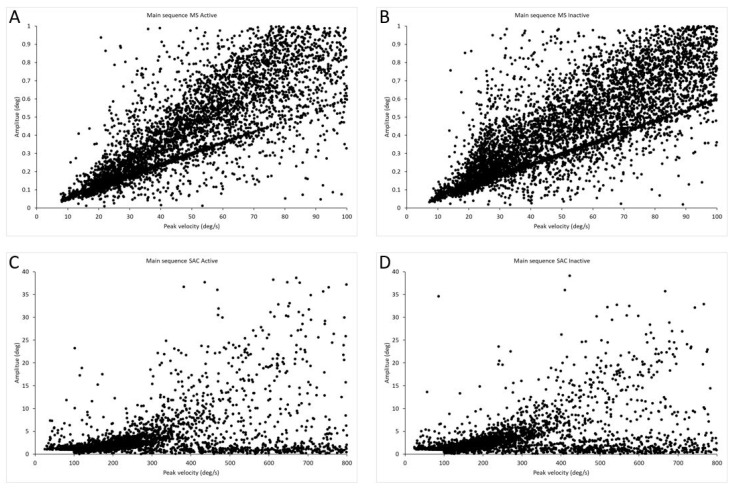
Main sequences for saccades and microsaccades. (**A**) Main sequence of microsaccades for active group, *n* = 5414. (**B**) Main sequence of microsaccades for inactive group, *n* = 6337. (**C**) Main sequence of saccades for active group, *n* = 3848. (**D**) Main sequence of saccades for inactive group, *n* = 4499.

Figure 3 shows the main sequence, i.e., the amplitude–peak velocity relationship of all microsaccades and saccades for active and inactive groups in all stimuli. As is visible in the figure, microsaccades follow the trend of large saccades. In the active group, the total number of microsaccades was 5414 and for saccades was 3848, while in the inactive group, the microsaccades were 6337 and saccades were 4499.

### 3.2. Microsaccades Rate

The main effect of stimulus showed significant differences (F_(3,69)_ = 2.84, *p* = 0.044, η^2^p = 0.106), suggesting that saccade rate differed across optic flow stimuli. Marginal means showed the highest rate in the baseline (1.25), with slightly lower rates for foveal, peripheral, and full-field optic flow (ranging from 1.03 to 1.11). The higher values were recorded in active males and the lowest in inactive females. All pairwise comparisons were not significant after Bonferroni correction, indicating that the overall effect reflects small but systematic differences.

**Figure 4:**
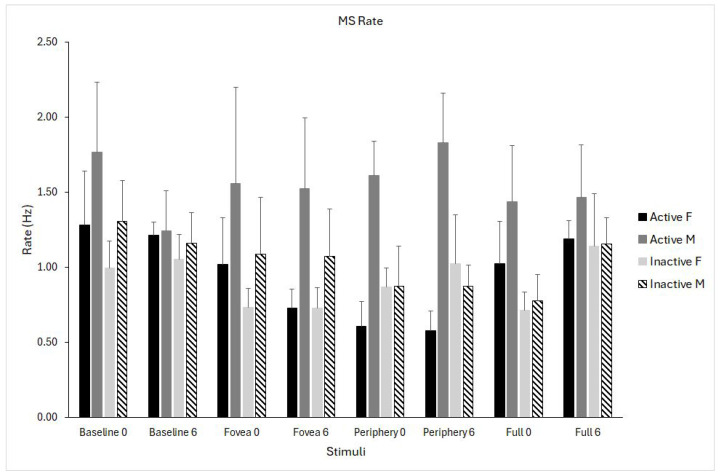
Microsaccade (MS) rate for the four optic flow stimuli (baseline, fovea, periphery, full field) measured at pre- (0) and post-intervention (6 months). Data are shown separately for physically active females (solid black), active males (solid grey), inactive females (light grey), and inactive males (dashed bars). Data are shown as means ± standard error (SE).

The stimuli × group × sex interaction showed significant differences (F_(3,69)_ = 4.662, *p* = 0.005, η^2^p = 0.163), indicating that the effect of visual stimulation on microsaccade rate differed as a function of both physical activity and sex (Figure 4).

### 3.3. Microsaccades Peak Velocity

**Figure 5:**
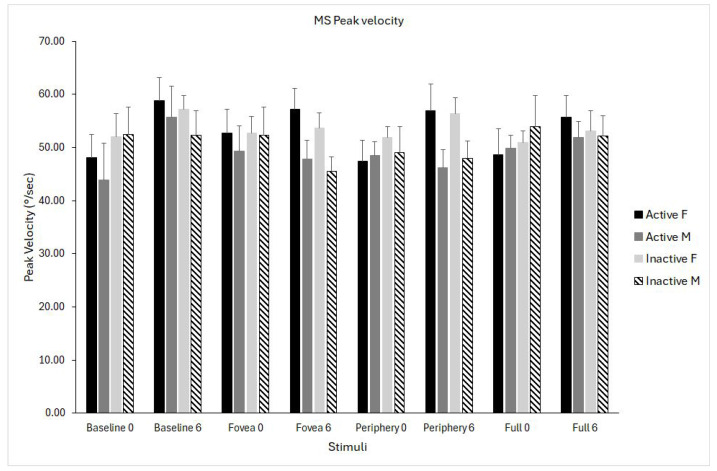
Microsaccade (MS) peak velocity for the four optic flow stimuli (baseline, fovea, periphery, full field) measured at pre- (0) and post-intervention (6 months). Data are shown separately for physically active females (solid black), active males (solid grey), inactive females (light grey), and inactive males (dashed bars). Data are shown as means ± standard error (SE).

The analysis revealed a significant interaction effect of time × stimulus (F_(1,24)_ = 3.08, *p* = 0.03, η^2^p = 0.114), indicating that changes in peak velocity from pre- to post-testing differed across visual conditions. Inspection of marginal means showed that peak velocity increased substantially in the baseline condition (+6.9 deg/s), whereas changes in the foveal, peripheral, and full-field optic flow conditions were minimal (between −0.7 and +2.7 deg/s) (Figure 5).

### 3.4. Microsaccades Amplitude

**Figure 6:**
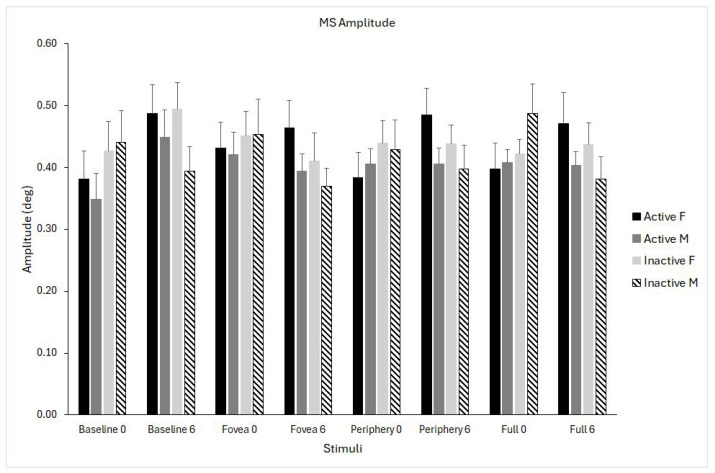
Microsaccade (MS) amplitude for the four optic flow stimuli (baseline, fovea, periphery, full field) measured at pre- (0) and post-intervention (6 months). Data are shown separately for physically active females (solid black), active males (solid grey), inactive females (light grey), and inactive males (dashed bars). Data are shown as means ± standard error (SE).

The time × stimulus interaction was significant (F_(1,24)_ = 3.502, *p* = 0.02, η^2^p = 0.127), reflecting modest differences in how amplitude changed over time depending on the stimulus. Inspection of marginal means suggested a small increase from pre- (ranging from 0.40 to 0.44°) to post-intervention (from 0.41 to 0.46°), with the pattern varying slightly across stimuli. The magnitude of these changes was small and consistent across participants (Figure 6).

## 4. Discussion

The present study aimed to examine whether habitual physical activity modulates microsaccade generation in individuals with T2DM during fixation under optic flow stimulation. This hypothesis was motivated by evidence that microsaccades are sensitive to attentional and oculomotor control, and that physical activity can influence cognitive–motor processes and oculomotor readiness. We further expected that optic flow, as a demanding visuomotor framework, would interact with physical activity level to reveal differences in microsaccade dynamics. In this context, variability was primarily observed in microsaccade rate, which showed differential modulation by optic flow across physical activity and sex groups, whereas amplitude and peak velocity exhibited smaller context-dependent changes over time. These findings extend existing microsaccade research by situating microsaccade dynamics within a metabolic condition and by examining their modulation by physical activity and optic flow context in individuals with T2DM.

### 4.1. Microsaccade Behaviour in T2DM

Microsaccades are highly sensitive indicators of oculomotor and attentional control [1,2,7]. Because diabetes is associated with microvascular, retinal, and neural complications that can affect fixation stability [9,21,22], we expected to find differences in microsaccade metrics as a function of physical activity level, which is known to influence cognitive-motor processes such as attention, arousal regulation, and oculomotor readiness [10,11,12]. Retinal microcirculatory alterations are a hallmark of diabetes and may affect visual processing even before overt retinopathy develops. While not directly assessed here, such microvascular changes could contribute to visuomotor alterations relevant to microsaccade control. The present finding is consistent with the view that cortical and subcortical circuits involved in microsaccade generation are modulated by physical exercise in individuals with T2DM who do not exhibit advanced retinopathy. Results showed that the total number of microsaccades was lower in the active group compared to the inactive one. The statistical analysis showed that only microsaccade rate showed a significant interaction effect of stimuli × group × sex, meaning that the effect of visual stimulation on microsaccade rate differed as a function of both physical activity and sex. Inspection of the interaction pattern indicated that microsaccade rate was highest in physically active males and lowest in inactive females, with intermediate values observed in active females and inactive males. These differences were most evident across optic flow conditions, suggesting that visual motion context amplified group- and sex-related modulation of microsaccade generation. This interaction highlights that microsaccade behaviour is shaped by a combination of physiological, behavioural, and sensory factors. In contrast, baseline fixation showed less pronounced separation between groups. The magnitude of these effects was moderate, indicating systematic but not extreme differences, consistent with inter-individual variability in attentional and oculomotor strategies rather than categorical impairment. Prior work indicates sex-dependent effects in oculomotor behaviour [31,32] and optic flow-based postural control [19], which may extend to fine oculomotor adjustments.

Previous studies indicate that oculomotor dysfunction becomes more pronounced in individuals with macular edoema, clinically relevant diabetic retinopathy, or peripheral neuropathy [9,21]. Our sample, characterised by mild to moderate metabolic impairment, may therefore represent a population in which subtle deficits in microsaccade generation do not yet manifest robustly during simple fixation tasks.

### 4.2. Temporal Changes in Peak Velocity and Amplitude

Microsaccade peak velocity and amplitude showed significant time × stimulus interactions, although the magnitudes of the changes were small. Such changes likely reflect context-dependent adjustments of fixational oculomotor control, potentially related to short-term adaptation or habituation to repeated visual stimulation. While measurement noise cannot be entirely excluded, the stimulus-specific nature of these effects suggests they are not random fluctuations. Although time × stimulus interactions were not explicitly hypothesised, their emergence is consistent with prior evidence of context-dependent modulation of microsaccade metrics and should be interpreted as exploratory indicators of dynamic oculomotor adjustment. Peak velocity increased specifically in the baseline condition, whereas optic flow conditions showed minimal modulation. Similarly, amplitude increased slightly from pre- to post-intervention across stimuli. These findings suggest that microsaccade metrics are not static but instead adapt to sensory context and repeated presentations. The stability of these measures across groups further supports the idea that, in subjects without diabetic retinopathy or advanced diabetic complications, fixational eye movements remain resistant to short-term behavioural variability. Importantly, this stability could depend on task demands, and microsaccade sensitivity may be greater during more complex or cognitively demanding fixation paradigms.

### 4.3. Physical Activity and Microsaccades: Why Were Group Differences Absent?

Beyond behavioural effects, physical activity may influence microsaccade generation through neurophysiological and cardiovascular–metabolic mechanisms. Regular aerobic exercise has been shown to promote neuroplasticity, enhance cerebral perfusion, and improve metabolic and vascular function [23], all of which are relevant to the integrity of oculomotor and attentional circuits. In individuals with diabetes, where microvascular dysfunction and altered neural processing may compromise visuomotor control, physical activity could therefore act as a modulatory factor supporting more efficient oculomotor regulation. Although these mechanisms were not directly assessed in the present study, they provide a plausible framework for interpreting the observed associations.

Despite consistent literature indicating improved attentional control and visuomotor performance in physically trained individuals [33,34,35], no main effects of physical activity emerged in this study. One possible explanation for the absence of robust group differences is that microsaccade generation relies on well-conserved brainstem and midbrain circuits [2], including the superior colliculus and associated saccadic burst generator pathways, which play a central role in initiating fixational eye movements. These circuits are known to operate with high temporal precision and may be relatively resistant to short-term behavioural or lifestyle-related modulation, particularly during simple fixation tasks. Thus, fine changes in attentional state or physical activity level may not be sufficient to substantially alter microsaccade dynamics at this level of control. A second explanation relates to the characteristics of the physically active participants in the present study. While regular physical activity was achieved, participants were not highly trained athletes. Previous research in sports and vision sciences has shown that activity-dependent modulation of eye movements, including microsaccades and saccades, is more consistently observed in individuals undergoing long-term, high-intensity, or cognitively demanding training regimes [10,11]. These findings suggest that more pronounced oculomotor adaptations may require higher training loads or task-specific visuomotor demands than those present in the current cohort.

The absence of robust microsaccade deficits in the present sample may also relate to the relatively preserved clinical profile of the participants. Although metabolic markers such as glycaemic control (HbA1c) and basic anthropometric measures were collected and are reported descriptively, disease duration and metabolic variables were not examined as formal moderators of microsaccade behaviour in the present analyses. Given evidence that visuomotor and oculomotor impairments in diabetes tend to emerge or worsen with longer disease duration and poorer metabolic control, future studies with larger samples should explicitly examine the moderating role of glycaemic status, disease chronicity, and related metabolic markers.

### 4.4. Limitations

The study was designed to focus on within-group variability among individuals with diabetes, examining how physical activity level, sex, and visual context modulate microsaccade behaviour rather than testing disease–control differences. As such, the findings should be interpreted as characterising relative modulation within a diabetic cohort. Future studies including matched healthy control groups and longitudinal designs will be important to determine the specificity and magnitude of diabetes-related alterations in microsaccade metrics.

Physical activity levels were assessed through self-reports, which may introduce recall or reporting bias; future studies could complement questionnaire-based measures with objective monitoring like accelerometry to further refine the characterisation of activity patterns in individuals with T2DM.

Although the number of trials per condition was limited, the large number of microsaccades recorded per participant supports the reliability of the estimated microsaccade metrics.

Even if age, BMI, and glycaemic control are relevant factors in diabetes-related visuomotor function, they were not included as covariates in the present analyses to avoid model overfitting; future studies with larger samples should explicitly examine their moderating effects.

### 4.5. Implications and Future Directions

The results of the present study demonstrate that microsaccade metrics are feasible, stable, and informative measures in individuals with T2DM. Optic flow processing is central to visuomotor integration, contributing to both gaze stabilisation and postural control through shared visual–oculomotor networks [36]. In this context, microsaccades recorded during optic flow viewing may reflect fine-scale oculomotor adjustments associated with the processing of visual motion cues relevant for postural regulation. Future studies are needed to further explore microsaccade characteristics, particularly rate, peak velocity, and amplitude, as sensitive functional indicators of diabetic retinal and oculomotor alterations, using longitudinal designs, retinal imaging, and multimodal motor and attentional assessments across disease stages.

Future developments in eye-tracking software, including automated microsaccade analysis and integration with behavioural or clinical data, could support the use of these metrics as standardised outcome measures in research and applied settings.

## 5. Conclusions

This study integrates microsaccade research, physical activity effects, and visuomotor characterisation in T2DM to examine how fine oculomotor control is modulated during optic flow viewing. By situating microsaccade dynamics within a metabolic condition and a demanding visuomotor context, the present findings contribute to understanding how physical activity and visual motion interact with oculomotor and attentional control in individuals with T2DM. Among the metrics examined, microsaccade rate, together with peak velocity and amplitude, emerged as the most sensitive to visual context and individual factors, suggesting their feasibility as outcome indicators in research-based interventions, such as exercise programmes, visuomotor training, or strategies aimed at improving metabolic control. More broadly, these results support the use of microsaccade metrics as complementary functional measures in diabetic oculomotor research, with potential value for probing subtle visuomotor alterations in early-stage or subclinical populations where standard clinical assessments may remain insensitive.

## Data Availability

The data that support the findings of this study are not openly available due to reasons of sensitivity and are available from the corresponding author upon reasonable request. Data are located in controlled access data storage at the University of Bologna (Italy).
